# Conditional Astroglial Rictor Overexpression Induces Malignant Glioma in Mice

**DOI:** 10.1371/journal.pone.0047741

**Published:** 2012-10-15

**Authors:** Tariq Bashir, Cheri Cloninger, Nicholas Artinian, Lauren Anderson, Andrew Bernath, Brent Holmes, Angelica Benavides-Serrato, Nesrin Sabha, Robert N. Nishimura, Abhijit Guha, Joseph Gera

**Affiliations:** 1 Department of Research and Development, Greater Los Angeles Veterans Affairs Healthcare System, Los Angeles, California, United States of America; 2 Department of Medicine, David Geffen School of Medicine, University of California Los Angeles, Los Angeles, California, United States of America; 3 Department of Neurology, David Geffen School of Medicine, University of California Los Angeles, Los Angeles, California, United States of America; 4 Labatt Brain Tumor Research Centre, Hospital for Sick Children Research Institute, University of Toronto, Toronto, Ontario, Canada; 5 Jonsson Comprehensive Cancer Center, University of California Los Angeles, Los Angeles, California, United States of America; 6 Molecular Biology Institute, University of California Los Angeles, Los Angeles, California, United States of America; Sanford Burnham Medical Research Institute, United States of America

## Abstract

**Background:**

Hyperactivation of the mTORC2 signaling pathway has been shown to contribute to the oncogenic properties of gliomas. Moreover, overexpression of the mTORC2 regulatory subunit Rictor has been associated with increased proliferation and invasive character of these tumor cells.

**Methodology/Principal Findings:**

To determine whether Rictor overexpression was sufficient to induce glioma formation in mice, we inserted a Cre-lox-regulated human Rictor transgene into the murine *ROSA26* locus. This *floxed* Rictor strain was crossed with mice expressing the *Cre* recombinase driven from the glial fibrillary acidic protein (GFAP) promoter whose expression is limited to the glial cell compartment. Double transgenic *GFAP-Cre/Rictor^loxP/loxP^* mice developed multifocal infiltrating glioma containing elevated mTORC2 activity and typically involved the subventricular zone (SVZ) and lateral ventricle. Analysis of Rictor-dependent signaling in these tumors demonstrated that in addition to elevated mTORC2 activity, an mTORC2-independent marker of cortical actin network function, was also elevated. Upon histological examination of the neoplasms, many displayed oligodendroglioma-like phenotypes and expressed markers associated with oligodendroglial lineage tumors. To determine whether upstream oncogenic EGFRvIII signaling would alter tumor phenotypes observed in the *GFAP-Cre/Rictor^loxP/loxP^* mice, transgenic *GFAP-EGFRvIII; GFAP-Cre/Rictor^loxP/loxP^* mice were generated. These mice developed mixed astrocytic-oligodendroglial tumors, however glioma formation was accelerated and correlated with increased mTORC2 activity. Additionally, the subventricular zone within the *GFAP-Cre/Rictor^loxP/loxP^* mouse brain was markedly expanded, and a further proliferation within this compartment of the brain was observed in transgenic *GFAP-EGFRvIII; GFAP-Cre/Rictor^loxP/loxP^* mice.

**Conclusion/Significance:**

These data collectively establish Rictor as a novel oncoprotein and support the role of dysregulated Rictor expression in gliomagenesis via mTOR-dependent and mTOR-independent mechanisms. Furthermore, oncogenic EGFRvIII signaling appears to potentiate the *in vivo* proliferative capacity of *GFAP-Cre/Rictor^loxP/loxP^* gliomas.

## Introduction

Despite combined therapeutic modalities, including surgery, radiation and chemotherapy, the prognosis for patients with malignant glioma, such as glioblastoma multiforme (GBM) remains poor with a median survival of only twelve months [1]. The appearance of *de novo* primary glioblastomas is associated with dysregulation of epidermal growth factor (EGFR) expression [2]–[4]. The most common EGFR mutation consists of an aberrantly spliced form which lacks exons 3 thru 6 (EGFRvIII) resulting in a constitutively activated receptor [5], [6]. Phosphoinositol-3-kinase (PI3K) signaling is also hyperactivated in most GBMs in association with EGFR mutation and/or PTEN tumor suppressor protein loss [7], [8].

Downstream of both oncogenic EGFR and PI3K signaling, the mTOR serine/threonine kinase exists in at least two complexes (mTORC1 & 2) which relay signals to distinct effectors [9]. PI3K mediated activation of mTORC1 via AKT, occurring due to inhibitory phosphorylation of the TSC1/TSC2 complex, links mTORC1 to PI3K signaling and the regulation of protein synthesis, cell size and proliferation [10]. mTORC2 responds to growth factor receptor activation, including EGFRvIII [11], and regulates cell survival, metabolism and the cytoskeleton [12]. mTORC1 specific components include Raptor and PRAS40, while the mTORC2 supracomplex uniquely contains Rictor, mSin1, Protor 1 and 2. Both mTORC1 and 2 contain mLST8, DEPTOR, the Tti1/Tel2 complex and the catalytic mTOR subunit [13].

Rictor, a 200 kD protein, was identified as a defining component of mTORC2 and does not exhibit significant sequence conservation between mammals and yeast, and lacks structural domains of known function [14]. The relative amount of Rictor which complexes with mTOR varies by cell type and inversely correlates with Raptor expression [14], [15]. Rictor binding to mTOR is unaffected by acute rapamycin exposure, however long term exposure results in reduced levels of complex abundance [16]. Rictor is also known to bind other protein partners, including the unconventional myosin motor Myo1C, the integrin linked kinase (ILK), Cullin-1, PKCς, Hsp70, and has demonstrated roles in the regulation of the cytoskeleton [14], [17]–[23].

Several reports have shown that the mTORC2 scaffolding protein Rictor is overexpressed in cancers including gliomas and has been shown to contribute to the oncogenic properties of these tumors, however, a causal role for Rictor in gliomagenesis has not been demonstrated [11], [22], [24]–[26]. We show here that glial fibrillary acidic protein Cre (GFAP-Cre) mediated conditional overexpression of Rictor is sufficient to induce intermediate-grade gliomas in mice. These oligodendroglial-like tumors exhibited elevated mTORC2 signaling *in vivo* and displayed increased growth, migratory capacity and invasiveness *in vitro*. Moreover, we demonstrate that cooperative signaling between *GFAP-EGFRvIII* and *GFAP-Cre/Rictor^loxP/loxP^* mice leads to hyperactivated mTORC2 signaling and results in high-grade gliomas.

## Results

### Generation of Rosa26hRictor^loxP/wt^ and GFAP-Cre/Rictor^loxp/loxp^ mice

**Figure 1:**
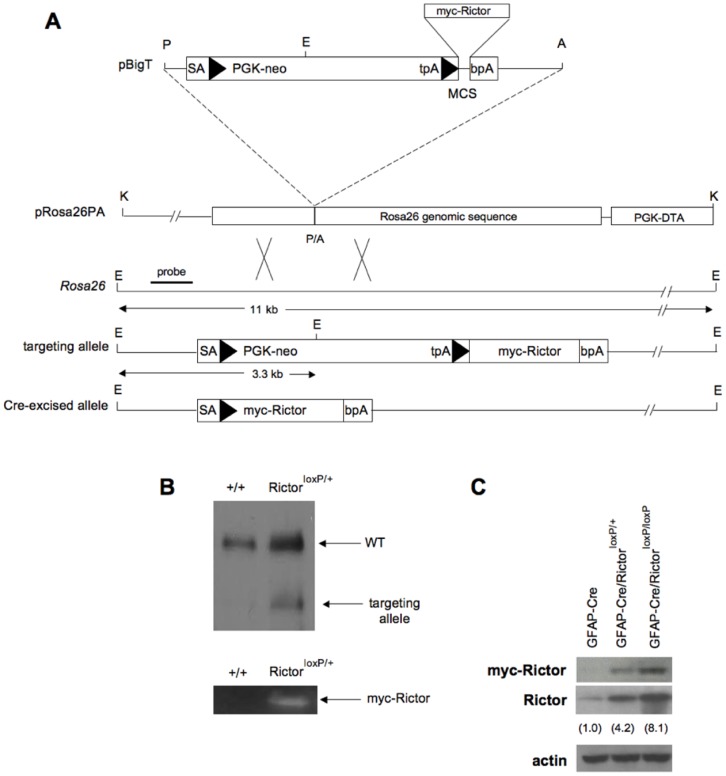
Generation of the ROSA26hRictor*^loxp/+^* mice (Rictor*^loxp/+^*). (A) Targeting strategy for Rictor*^loxp/+^*. A human myc-tagged Rictor sequence was cloned into the pBigT plasmid and subcloned into the targeting vector pROSA26PA. The predicted sizes of EcoRV (E) – digested DNA fragments are shown when hybridized to the indicated probe. (B). Southern blot of EcoRV-digested genomic DNA from +/+ or Rictor*^loxp/+^*mice hybridized to the probe shown in (A). In the lower panel, genomic PCR-based detection of the myc-Rictor transgene sequences is shown in +/+ and Rictor*^loxp/+^* mice. (C) Immunoblot of brain extracts from GFAP-Cre, GFAP-Cre/Rictor*^loxp/+^*, or GFAP-Cre/Rictor*^loxp/loxp^* mice using antibodies against the myc-tagged Rictor demonstrating Cre-dependent expression of Rictor. Values in parentheses are fold increases in total Rictor expression relative to endogenous expression levels in GFAP-Cre mice as determined by densitometric analysis of an immunoblot using antibodies reactive to both human and murine Rictor.

To examine whether Rictor overexpression would induce tumor formation *in vivo* we generated a mouse model in which a myc-tagged human Rictor transgene was conditionally expressed in glial cells. Using homologous recombination in embryonic stem cells, the Rictor transgene was inserted into the *ROSA26* locus downstream of a floxed PGK-neo cassette containing a strong transcriptional termination stop sequence (Figure 1A) [27]. The transgene was not expressed unless Cre-mediated recombination resulted in removal of the cassette and allowed expression of Rictor. Southern blot and PCR analysis confirmed appropriate targeting of the transgene (Figure 1B) and Cre-dependent over-expression of the Rictor allele in heterozygous and homozygous animals was assessed by immunoblot (Figure 1C). Heterozygotes were intercrossed to obtain litters of pups with Mendelian distributions of the F2 genotypes. Glial cell restricted expression of the Cre recombinase was the result of matings to the GFAP-Cre mouse line developed in Albee Messing's laboratory [28]. This line demonstrates widespread recombinase activity in neurons, glia and GFAP positive subventricular zone (SVZ) precursor cells in adult GFAP-Cre mouse brain [28], [29].

### Glioma formation and elevated mTORC2 activity in GFAP-Cre/Rictor^loxP/loxP^ mice

**Figure 2:**
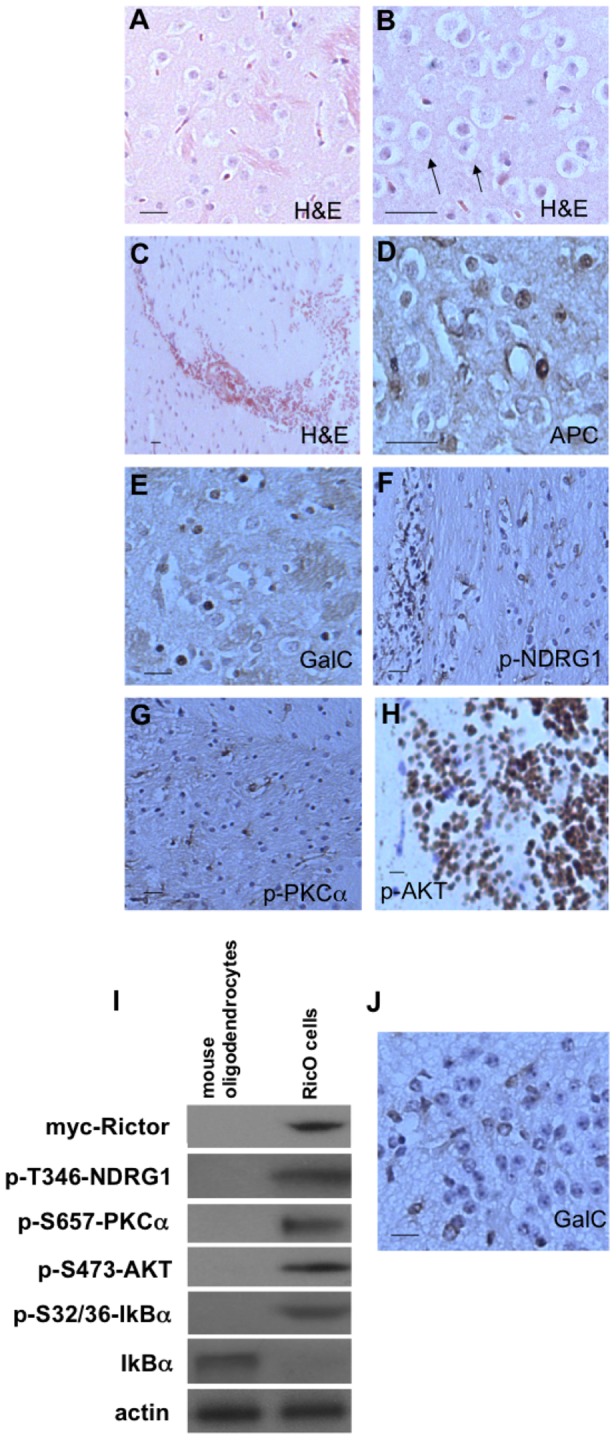
Gliomas in GFAP-Cre/Rictor*^loxp/loxp^* transgenic mice. (A) Low magnification of H & E-stained multifocal gliomas. (B) H & E-stained oligodendroglial tumor cells. (C) Representative gliomas displaying chicken wire-like vascularity observed in oligodendrogliomas. (D&E) Oligodendroglial tumor cells immunoreactive for APC and GalC, respectively. (F–H) Sections were stained with antibodies to phospho-T^346^-NDRG1, phospho-S^657^-PKCα and phospho-S^473^-AKT, respectively and demonstrated increased staining in oligodendroglial cells. In A–H, scale bar; 20 µm. (I) Increased mTORC2 signaling determined by immunoblot of Ric0 oligodendroglial cell line established from a newborn GFAP-Cre/Rictor*^loxp/loxp^* glioma. The Rictor transgene was detected using antisera specific for human Rictor. (J) GalC immunoreactive Ric0 cells demonstrating oligodendroglial lineage. Scale bar; 20 µm.

Offspring from both heterozygous (Rictor*^loxP/+^*) and homozygous (Rictor*^loxP/loxP^*) crosses to the GFAP-Cre line were born in the expected Mendelian ratios and appeared normal at birth. Upon examination of the brain at the time of euthanasia no macroscopic abnormalities were found, however, in GFAP-Cre/Rictor*^loxP/loxP^* animals histological examination revealed bilateral, multifocal infiltrating glioma with nearly complete penetrance. In several cases, tumors involved the amygdalohippocampal region or surrounding cortex and were also found within close proximity to the subventricular zone (SVZ). The gliomas were characterized at low magnification by foci of hypercellularity on H & E stain (see Figure 2A). Infiltrating glioma cells exhibited oligodendroglial-like features with tumor cells displaying the typical fried egg appearance (Figure 2B). In addition, infiltrative tumors had regions of intratumoral hemorrage with chicken wire-like vascularity seen in human oligodendrogliomas (Figure 2C) [30], [31]. The infiltrating growth pattern, degree of hypercellularity and atypism seen in these tumors were consistent with intermediate grade II–III gliomas based on WHO criteria [32]. Many infiltrating cells were APC and GalC immunoreactive (Figure 2D&E, oligodendrocyte markers) staining positive for phospho-T^346^-NDRG1, phospho-S^657^-PKCα, phospho-S^473^-AKT, phospho-S^32/36^-IkBα, markers of mTORC2 activity (Figure 2F–H). Additionally, in a cell line (Ric0) derived from resected gliomas from newborn *GFAP-Cre/Rictor^loxP/loxP^* mice expressed the Rictor transgene, stained positive for GalC and demonstrated hyperactivation of mTORC2 as compared to oligodendrocytes isolated from control littermates (Figure 2I–J).

### mTORC2-independent functions of Rictor are increased in GFAP-Cre/Rictor^loxP/loxP^ mice

**Figure 3:**
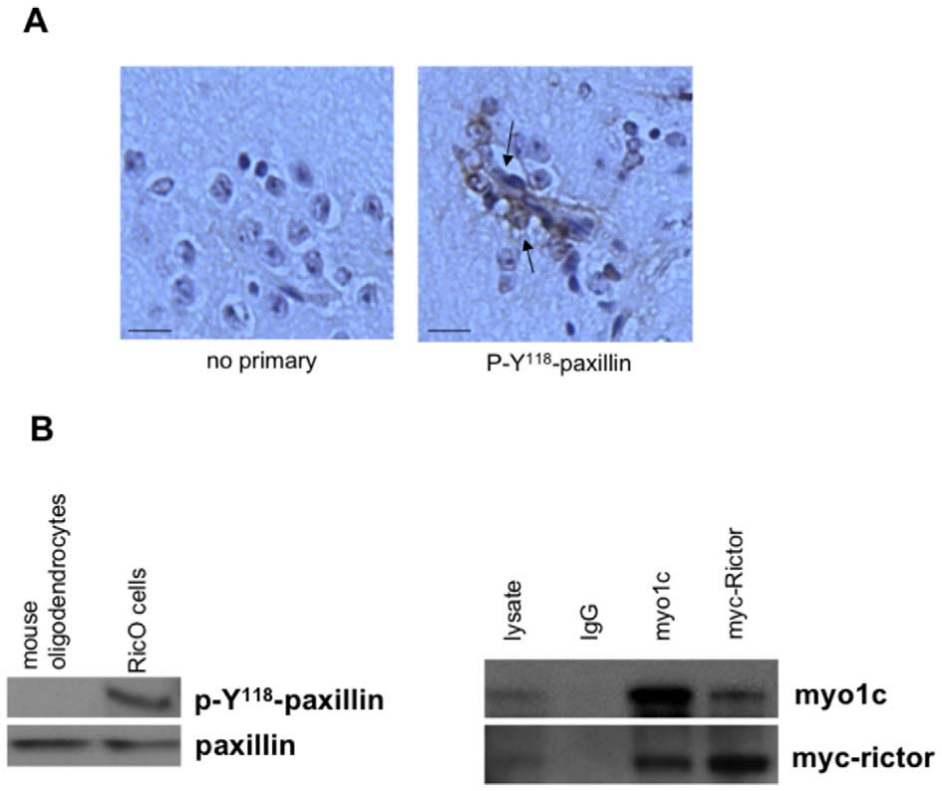
GFAP-Cre/Rictor*^loxp/loxp^* gliomas demonstrate mTORC2-independent signaling. (A) Elevated phospho-Y^118^-paxillin staining in GFAP-Cre/Rictor*^loxp/loxp^* gliomas. Left panel, no primary antibody; right panel, anti-phospho-Y^118^-paxillin antibody. Scale bar; 20 µm. (B) Elevated phospho-Y^118^-paxillin levels in Ric0 cells as determined by immunoblot analysis (left panel); co-immunoprecipitation of the myc-tagged Rictor-Myo1C complex in Ric0 cells (right panel). Whole cell lysate was immunoprecipitated with the indicated antibodies and immunoprecipitates were immunoblotted for either Myo1C or the myc-tag.

Several reports have implicated Rictor in functions distinct from its role within mTORC2 [19], [20], [22], [23]. Rictor has been shown to directly interact with the unconventional myosin Myo1c to form a complex which regulates the cortical actin cytoskeletal network [19]. Tyrosine 118 phosphorylation of paxillin is associated with the regulation of dynamic cortical actin events and requires the Myo1c-Rictor complex [19], [33]. Moreover, this phosphorylation event occurs independent of mTOR or PI3K signaling [19]. To address whether gliomas from *GFAP-Cre/Rictor^loxP/loxP^* mice contained elevated levels of phospho-Y^118^-paxillin sections were stained via immunohistochemistry using phospho-specific antibodies. As shown in Figure 3A, phospho-Y^118^-paxillin positive cells were detected in many oligodendroglial cells suggesting that Rictor overexpression may indeed promote Myo1C-Rictor/paxillin signaling. Similarly, in Ric0 cells, phospho-Y^118^-paxillin levels were elevated compared to purified oligodendrocytes from control littermate mice (Figure 3B, left panel). We were also able to detect the endogenous Myo1C-Rictor complex via coimmunoprecipitation in Ric0 cells. As shown in the right panel of Figure 3B, Myo1C antibodies coimmunoprecipitated myc-tagged Rictor, as well as, in the reciprocal coimmunoprecipitation reaction using anti-myc to detect Myo1C. These data taken together strongly suggest that in gliomas from *GFAP-Cre/Rictor^loxP/loxP^* mice Rictor participates in mTORC2-independent activation of cortical actin dynamics.

### Cooperative signaling between EGFRvIII and mTORC2 in mice results in accelerated glioma development

**Figure 4:**
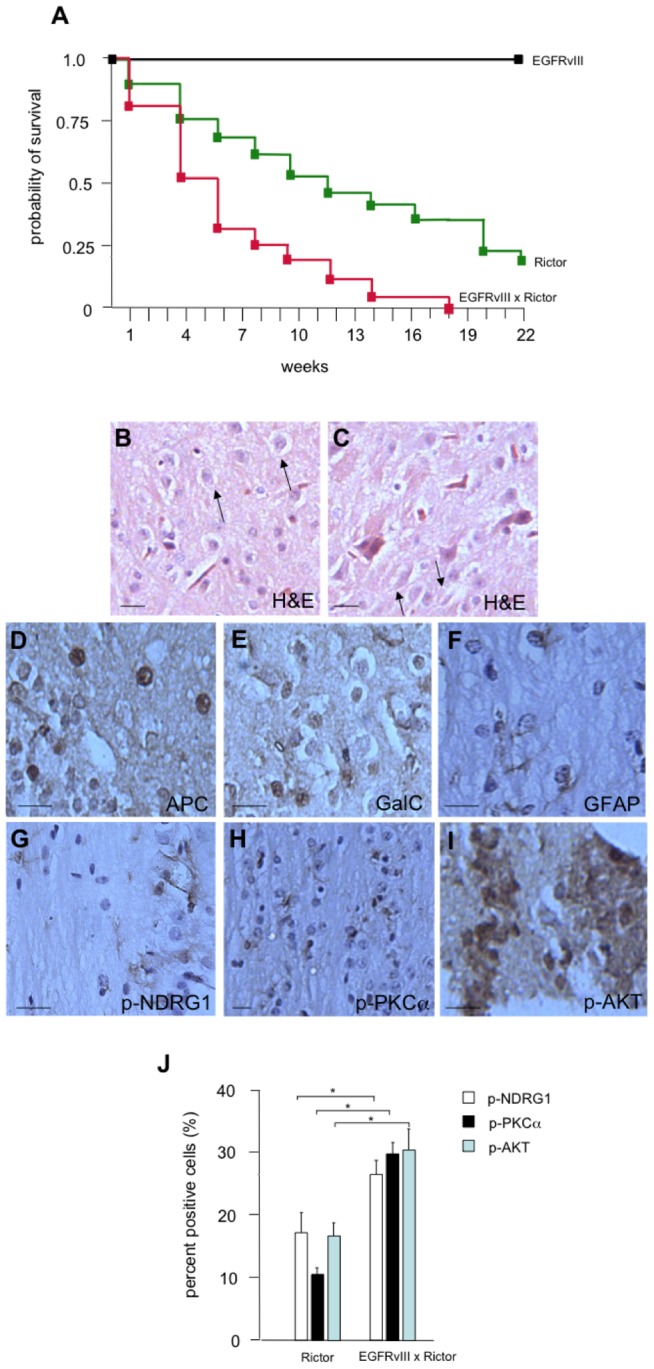
Transgenic *GFAP-EGFRvIII; GFAP-Cre/Rictor^loxP/loxP^* mice exhibit reduced survival, enhanced tumor grade and mTORC2 signaling. In (A), wild-type mice had 100% tumor-free survival in these experiments (not shown), as did GFAP-EGFRvIII transgenic mice. Twenty-six transgene-positive mice were followed for twenty-two weeks (EGFRvIII). Twenty-nine transgene-positive *GFAP-Cre/Rictor^loxP/loxP^* mice were followed for twenty-two weeks (Rictor). Transgene-positive *GFAP-EGFRvIII* and *GFAP-Cre/Rictor^loxP/loxP^* mice were crossed and a cohort of twenty-four animals were followed for eighteen weeks (EGFRvIII x Rictor). (B&C) Increased grade of oligodendroglioma in *GFAP-EGFRvIII; GFAP-Cre/Rictor^loxP/loxP^* mice. H & E sections of a typical tumor displayed increased regions of necrosis and cells with pyknotic nuclei. Increased endothelial proliferation was also observed consistent with anaplastic oligodendroglioma in humans (grade 3 WHO). (D–F) APC, GalC and GFAP immunoreactive cells from representative oligodendroglial tumors. (G–I) mTORC2 markers displayed elevated immunohistochemical staining in sections of gliomas from *GFAP-EGFRvIII; GFAP-Cre/Rictor^loxP/loxP^* mice. In B–I, scale bar; 20 µm. (J) Quantification of mTORC2 signaling markers in gliomas from *GFAP-Cre/Rictor^loxP/loxP^* and *GFAP-EGFRvIII; GFAP-Cre/Rictor^loxP/loxP^* mice. A representative count in triplicate out of three individual counts with similar results is shown with + S.D. *, *P*<0.05.

**Figure 5:**
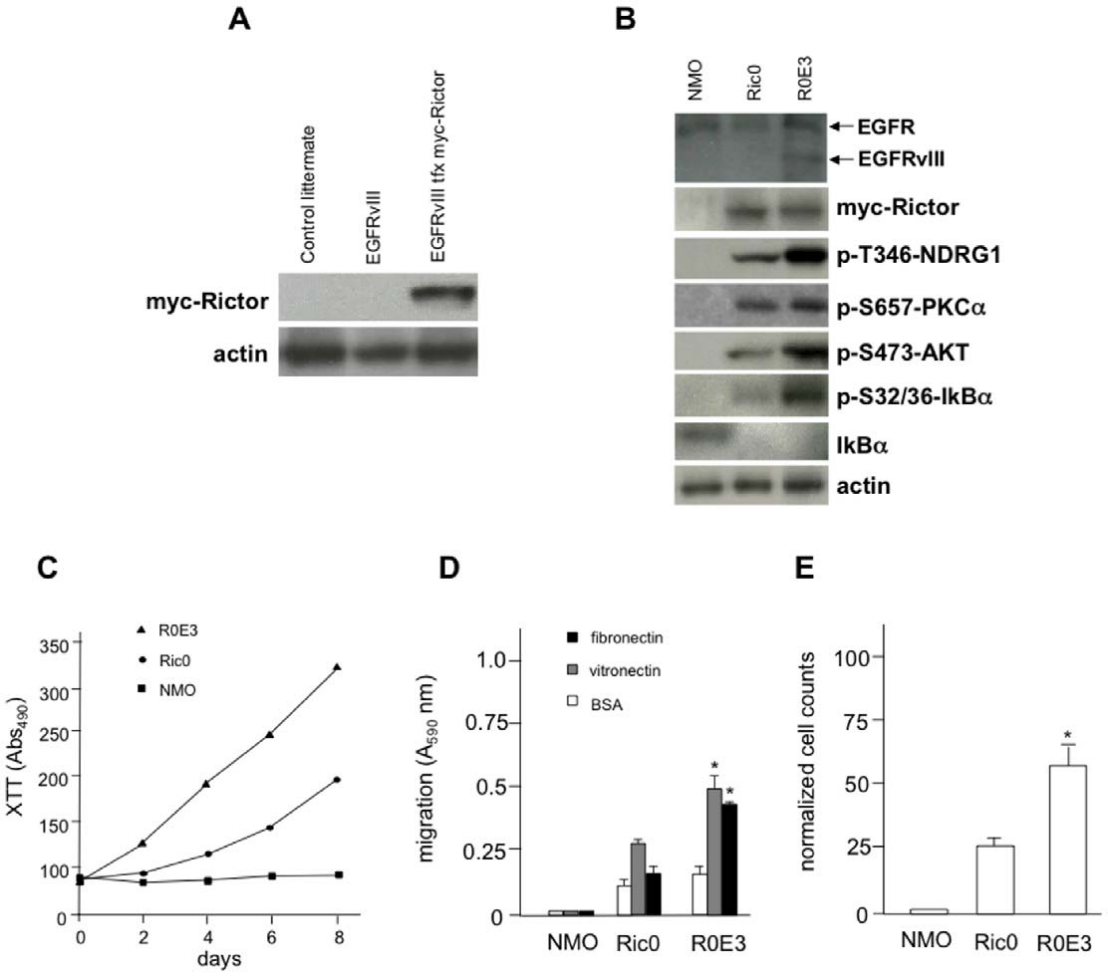
Cooperative signaling between oncogenic EGFR and mTORC2 results in enhanced gliomagenesis *in vitro*. (A) Immunoblot analysis for myc-tagged Rictor in oligodendrocytes obtained from control littermates, *GFAP-EGFRvIII, or GFAP-EGFRvIII; GFAP-Cre/Rictor^loxP/loxP^* mice. (B) Cell line established from *GFAP-EGFRvIII; GFAP-Cre/Rictor^loxP/loxP^* oligodendroglioma (R0E3) displays elevated mTORC2 signaling in comparison to Ric0 cells. Lysates of normal mature oligodendrocytes (NMO), Ric0 and R0E3 cells were immunoblotted for the indicated proteins. (C) XTT proliferation assay of established normal mature oligodendrocytes (squares), transgenic Ric0 (circles) and R0E3 (triangles) cells. (D) Motility of NMO, Ric0 and R0E3 cells in Boyden chamber assays migrating towards, BSA (white bars), vitronectin (grey bars) or fibronectin (black bars), (*, *P*<0.05) (E) Invasive potential of NMO, Ric0 and R0E3 cells in matrigel invasion assay (*, *P*<0.05). Counts were normalized to the viable cell count to exclude any effects of increased cell number on transmigration. The data shown in (D&E) are the mean + S.D. of three independent experiments.

To determine whether their existed a potential interaction between oncogenic EGFRvIII and Rictor proteins, we crossed *GFAP-Cre/Rictor^loxP/loxP^* mice with a previously characterized transgenic strain which expresses the constitutively active EGFRvIII allele driven from the GFAP promoter [34]. In addition to genotyping to confirm the presence of the transgenes in these mice, derived oligodendroglial tumor cultures from these transgenic mice brain (R0E3 cells) showed expression of EGFRvIII protein by immunoblot analysis. Moreover, immunoblot analysis confirmed Y^1068^-EGFRvIII phosphorylation, as well as specific myc-Rictor transgene expression only in *GFAP-EGFRvIII; GFAP-Cre/Rictor^loxP/loxP^* mice (see Supplementary Figure 2 and Figures 5A&B). As shown in Figure 4A, GFAP-EGFRvIII mice (hemizygous and homozygous) were healthy and did not demonstrate any clinical abnormalities as previously described [34]. In homozygous *GFAP-Cre/Rictor^loxP/loxP^* mice 50% of animals developed tumors consistent with Grade I–II histopathology by 12–14 weeks and with 75% of animals succumbing to gliomas by 22 weeks. Transgenic *GFAP-EGFRvIII; GFAP-Cre/Rictor^loxP/loxP^* mice exhibited a marked reduction in overall survival with 50% of animals developing gliomas by 4–6 weeks and all of the mice succumbing by 18 weeks. The histopathology of the tumors from *GFAP-EGFRvIII; GFAP-Cre/Rictor^loxP/loxP^* mice was more advanced and consistent with higher-grade mixed astrocytic-oligodendroglial lineage (Grade II–III, Figure 4B&C) as compared to tumors from the *GFAP-Cre/Rictor^loxP/loxP^* mice (Figure 2A&B). *GFAP-EGFRvIII; GFAP-Cre/Rictor^loxP/loxP^* glial tumors were strongly APC and GalC positive by immunohistochemical staining (Figure 4D&E) and to a lesser extent GFAP positive consistent with a mixed astrocytic-oligodendroglial tumor. These tumors also displayed elevated mTORC2 signaling and stained positive for phospho-T^346^-NDRG1, phospho-S^657^-PKCα, phospho-S^473^-AKT shown in Figures 4G–I, respectively. Moreover, as shown in Figure 4J, quantification of the immunohistochemical staining results comparing relative mTORC2 marker levels in tumors from *GFAP-EGFRvIII; GFAP-Cre/Rictor^loxP/lox^* mice to those from *GFAP-Cre/Rictor^loxP/loxP^* mice demonstrated significantly greater numbers of immuno-positive cells for all three markers in *GFAP-EGFRvIII; GFAP-Cre/Rictor^loxP/lox^* tumors. We also examined the *in vitro* signaling and oncogenic properties of the derived tumors cell lines from both *GFAP-Cre/Rictor^loxP/loxP^* and *GFAP-EGFRvIII; GFAP-Cre/Rictor^loxP/loxP^* glial tumors. As shown in Figure 5B, while the total amount of myc-Rictor expressed from the transgene was comparable in the Ric0 and R0E3 cells, the mTORC2 markers phospho-T^346^-NDRG1, phospho-S^657^-PKCα, phospho-S^473^-AKT, phospho-S^32/36^-IkBα demonstrated elevated levels in R0E3 cells. Total IkBa levels were also undetectable in Ric0 or R0E3 cells consistent with its observed phosphorylation status. We also examined the relative growth, migration and invasive characteristics of the Ric0 and R0E3 lines *in vitro*. As shown in Figure 5C, R0E3 cells grew approximately twice as fast as Ric0 cells as determined by XTT proliferation assays. To assess whether cell migration was also enhanced in R0E3 cells we compared the ability of cells to traverse either a vitronectin-coated or fibronectin-coated Boyden chamber utilizing bovine serum albumin (BSA)-coated chambers as a control. We additionally tested whether R0E3 cells showed increased invasive properties as determined by their ability to invade Matrigel. As shown in Figures 5D&E, R0E3 cells derived from *GFAP-EGFRvIII; GFAP-Cre/Rictor^loxP/loxP^* glial tumors demonstrated increased motility as compared to Ric0, *GFAP-Cre/Rictor^loxP/loxP^* -derived glial tumors. To demonstrate that both the Ric0 and R0E3 were neoplastic and not dysplastic cells, we also determined whether they would form tumors in xenografted SCID mice. Tumors developed with both lines in recipient mice within 2–3 weeks (data not shown) with histological characteristics similar to the original gliomas from the respective transgenic mice (see supplementary Figure 3). These data taken together strongly suggest a cooperative interaction between oncogenic EGFRvIII and Rictor *in vivo* to promote gliomagenesis via increases in mTORC2 activity.

### GFAP-Cre/Rictor^loxP/loxP^ and GFAP-EGFRvIII; GFAP-Cre/Rictor^loxP/loxP^ mice display marked subventricular zone expansion

**Figure 6:**
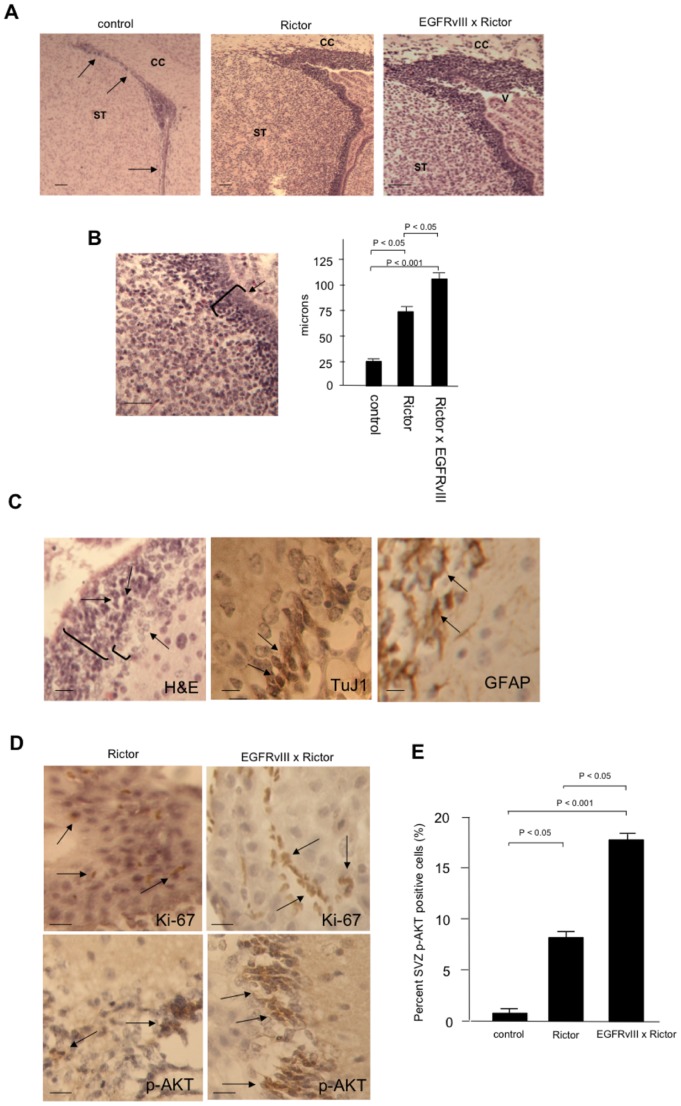
Expansion of the subventricular zone in *GFAP-Cre/Rictor^loxP/loxP^* and *GFAP-EGFRvIII; GFAP-Cre/Rictor^loxP/loxP^* mice. (A) Parasagittal sections of the SVZ from control, *GFAP-Cre/Rictor^loxP/loxP^* (Rictor) and *GFAP-EGFRvIII; GFAP-Cre/Rictor^loxP/loxP^* (Rictor x EGFRvIII) mice. Arrows show the SVZ/rostral migratory stream in mature control mouse brain; ST, striatum; CC, corpus callosum; V; ventricle. Scale bar, 100 µm. (B) The SVZ was measured at its single widest point (left panel, representative measurement; arrow) and quantified. Five mice of each strain were examined. (C) Expanded SVZ in *GFAP-Cre/Rictor^loxP/loxP^* mice are composed of distinct cell populations. Representative adjacent sections stained with H&E, immunohistochemically with anti-TuJ1 and anti-GFAP antibodies as indicated. Similar results were observed in SVZ sections in *GFAP-EGFRvIII; GFAP-Cre/Rictor^loxP/loxP^* mice. (D) Representative SVZ sections stained immunohistochemically with anti-Ki-67 and anti-phospho-S^473^-AKT antibodies from *GFAP-Cre/Rictor^loxP/loxP^* (Rictor) and *GFAP-EGFRvIII; GFAP-Cre/Rictor^loxP/loxP^* (Rictor x EGFRvIII) mice as indicated. In B-D, scale bar; 20 µm. (E) Quantification of phospho-S^473^-AKT immunopositive cell numbers in the SVZ of control, *GFAP-Cre/Rictor^loxP/loxP^* (Rictor) and *GFAP-EGFRvIII; GFAP-Cre/Rictor^loxP/loxP^* (Rictor x EGFRvIII) mice. A representative count in triplicate out of three individual counts with similar results is shown with + S.D.

There are examples demonstrating the ability of several mutated receptor tyrosine kinases, as well as tumor suppressor loss, to converge on the mTOR pathway to enhance gliomagenesis *in vivo* [35]–[38]. Moreover, recent studies demonstrate that in the *Nf1*-loss model of glioma in mice, mTORC2 activity promotes neural stem cell proliferation and expansion in a manner dependent on Rictor expression [39]. The most extensively characterized neural stem cell compartment resides in the subventricular zone (SVZ) [40]–[43], thus we determined whether this region displayed alterations in the transgenic mice. In both *GFAP-Cre/Rictor^loxP/loxP^ and GFAP-EGFRvIII; GFAP-Cre/Rictor^loxP/loxP^* mice the subventricular zone was markedly thickened, with mean thickness increases of approximately 2- and 3-fold, respectively, as compared to age-matched control transgene-negative littermates (Figure 6A&B). Most of the expanded cells were morphologically similar with oval to angular nuclei, containing little cytoplasm and were not immunoreactive for GFAP, however were TuJ1 positive (early neuronal differentiation marker) (Figure 6C, center panel). Apoptotic bodies were observable, as well as mitotic figures (Figure 6C, left panel). We also noted a second cell type that was larger, contained increased cytoplasm and many of these cells stained immuno-positive for GFAP (Figure 6C, right panel). Cells within the expanded SVZ stained positive for Ki-67 and phospho-S^473^-AKT in both *GFAP-Cre/Rictor^loxP/loxP^ and GFAP-EGFRvIII; GFAP-Cre/Rictor^loxP/loxP^* mice (Figure 6D) and a greater number of SVZ phospho-S^473^-AKT immunopositive cells were observed in the *GFAP-EGFRvIII; GFAP-Cre/Rictor^loxP/loxP^* mice compared with age-matched *GFAP-Cre/Rictor^loxP/loxP^* mice (Figure 6E). These data suggest that *in vivo* expression of Rictor alone is sufficient to result in marked expansion of the SVZ and correlates with elevated mTORC2 activity in these cells. Furthermore, cooperative interactions between Rictor and oncogenic EGFRvIII signaling can potentiate SVZ cell expansion consistent with increased numbers of cells containing activated mTORC2.

## Discussion

Several studies have described the overexpression of Rictor in a variety of cancers including gliomas [11], [24]–[26], as well as, its ability to function as a haploinsufficiency gene protecting *Pten^+/−^* heterozygous mice from prostate cancer [44]. Our previous study demonstrated that Rictor overexpression promoted the oncogenic properties of GBM lines and as a prelude to formal transgenic studies we also examined whether overexpression of Rictor was sufficient to induce carcinogenesis in the classic NIH3T3-focus forming assay (see supplementary Figure 1A). Rictor overexpression induced focus formation and additionally cooperated with activated H-*ras*V^12^ to increase transformed focus formation in *Ink4a/Arf*-deficient MEFs (supplementary Figure 1B). These observations provided the impetus for us to examine whether Rictor overexpression would induce gliomagenesis in the mouse. In this report, we describe the generation and characterization of transgenic mice with conditional overexpression of Rictor in astroglial cells resulting in multifocal-intermediate and low-grade glioma. Moreover, we demonstrate that mice derived from crosses between these overexpressors and those carrying a GFAP-driven constitutively active EGFR variant transgene harbor more aggressive, higher-grade gliomas consistent with hyperactivated mTORC2 signaling.

*GFAP-Cre/Rictor^loxP/loxP^* mice developed gliomas reminiscent of human oligodendroglioma staining immunopositive for APC and GalC. We also observed regions of satellitosis and more rarely, subpial accumulation of tumor cells often observed in oligodendrogliomas. Tumor cells from this strain exhibited immunoreactivity to known mTORC2 substrates demonstrating increased mTORC2 activity as a direct result of Rictor overexpression. These data are consistent with the notion that increased Rictor levels are sufficient to lead to elevated nucleation of mTORC2 resulting in a larger pool of supracomplexes which are competent to transduce signal. mTOR immunoprecipitations performed on Ric0 cells support this hypothesis (supplementary figure 3B). Additionally, we observed increased mTORC2-independent signaling effects of Rictor overexpression in gliomas from *GFAP-Cre/Rictor^loxP/loxP^* mice and in cell lines established from these tumors (see Figure 3). These data suggest that in addition to stimulating mTORC2, Rictor may also impinge on other signaling pathways. One such pathway may be downstream activation of the Rho family of GTPases [45]. The *S. cerevisiae* homolog of Rictor, AVO3 is known to contain a RasGEFN domain [17]. This domain exists N-terminal to the catalytic GDP/GTP exchange domain in some guanine nucleotide exchange factors such as the Ras-like small GTPases [46]. Thus, it is possible that Rictor may mediate activation of GTPase signaling.

We observed significant expansion of the SVZ in *GFAP-Cre/Rictor^loxP/loxP^* mice and this was even more pronounced in the *GFAP-EGFRvIII; GFAP-Cre/Rictor^loxP/loxP^* transgenic strain. Our data suggested that both GFAP and TuJ1-expressing cell populations within the SVZ from both transgenic strains were present in this compartment and likely participate in the observed proliferation. Current data suggests that type B cells within the SVZ (GFAP positive cells) are the actual primary progenitors within this compartment which give rise to actively proliferating transit amplifying, which give rise to immature neuroblasts (TuJ1 positive) [41]. The expansion of the SVZ in our transgenic strains is similar to those observed in transgenic mice expressing GFAP-Cre^+^/*K-ras^G12D^* as well as in wild-type mice receiving intraventricular infusions of EGF or PDGF [47]–[49]. Our data suggest that elevated Rictor levels alone or in cooperation with upstream EGFRvIII overexpression leads to proliferation in the SVZ and may constitute an originating stem cell in these transgenic strains, however we cannot rule out contributions from other germinal regions of the brain.

Our results are also consistent with the observation that AKT activity is critical initiator of gliomagenesis. Previous reports have demonstrated that pharmacological inhibition of the PI3K/AKT axis reduces the number of GFAP-expressing cells in the olfactory bulb [50]. Additionally, abrogating PI3K/AKT signaling alters the subcellular localization of Olig2 which is required for astrocyte differentiation [51]. The mTOR complex containing Rictor positively regulates AKT activity via phosphorylation on serine 473 and recent data suggests that variable Rictor expression levels may also regulate brain region specific stem cell proliferation and gliomagenesis in a manner dependent on selective AKT hyperactivity [39].

In summary, our results demonstrate that Rictor overexpression alone is sufficient to produce gliomas which resemble human oligodendroglial tumors in the mouse. Moreover, oncogenic EGFRvIII signaling cooperates with Rictor overexpression to induce more aggressive mixed astrocytic-oligodendroglial tumors. *GFAP-Cre/Rictor^loxP/loxP^* tumors harbor elevated mTORC2 activity which is further induced in tumors from *GFAP-EGFRvIII; GFAP-Cre/Rictor^loxP/loxP^* mice. Finally, hyperactive mTORC2 signaling in cells of the SVZ results in marked expansion of this stem cell compartment suggesting that these cells may constitute the glioma cell of origin in these models. These models may provide future insights into the mechanistic basis of gliomagenesis and resistance to chemotherapeutic intervention.

## Materials and Methods

### Vector Construction and ES cell transgenics

The human myc-tagged Rictor was obtained from Dr. David Sabatini (MIT, Cambridge, MA) and cloned into the shuttling vector pBigT and subsequently subcloned into *ROSA26PA* (kind gift of Dr. Frank Costantini, Columbia University, NY) [27]. 129ES cells were electroporated and grown without feeders under selection in 300 mg/ml G418 for 7 days. 129ES cells (Dr. Hong Wu, UCLA) were derived from 129/Sv mice using standard methods [52]. Clones were isolated and screened by genomic PCR and Southern hybridization. The 5′ UTR probe used detects an 11 kb wild-type band and a 3.8 kb targeted band.

### Mice and Genotyping

*Rosa26hRictor^loxP/wt^* mice were generated following a protocol similar to that described previously [27]. Mice were bred on a mixed 129/Sv-FVB/N genetic background. *GFAP-EGFRvIII* mice, originally on an outbred ICR background were backcrossed at least eight generations into FVB/N inbred mice. Genotyping of the *GFAP-Cre^+^* [FVB-Tg(GFAP-Cre)25Mes/J; The Jackson Laboratory] and *GFAP-EGFRvIII* mice was performed as previously described [28], [34]. Genotyping of *Rosa26hRictor^loxP/wt^* mice was as described for *Gt(ROSA)26Sor^tm1Sor^* with minor modification [53]. The primers used were as follows: RF216, 5′- GTGGTGGTGGGTCGACGATGG -3′; RR39, 5′-AAGCGCTCGTAGCCCTGCTG -3′. All protocols conformed to the guidelines established by the Association for the Assessment and Accreditation of Laboratory Animal Care and were approved by the UCLA-VA Animal Care and Use Committees under protocol numbers 0010-11036 and 0009-09029.

### Histology and Immunohistochemistry

Tissue for histological analysis was immersion-fixed in buffered formalin, paraffin-embedded, sectioned and stained with H&E according to established protocols. Immunohistochemical analysis was performed according to standard procedures with antigen retrieval performed as previously described [54]. Samples were blocked in either 20% goat serum or 10% rabbit serum in PBS, followed by overnight incubation in primary antibody at 4°C. The primary antibodies used were as follows: rabbit anti-GFAP (Dako, Carpinteria, CA), rabbit anti-GalC (Proteintech, Chicago, IL), Anti-APC (Ab-7, EMD Millipore, Billerica, MA), phospho-T^346^- NDRG1, phospho-S^657^-PCKα, phospho-S^473^-AKT (Cell Signaling, Danvers, MA), rabbit anti-phospho-Y^118^-paxillin (GenWay, San Diego, CA), Ki-67 (Vector Laboratories, Burlingame, CA), TUJ1 (Covance, Vienna, VA). Quantification of immunopositive cell numbers was performed on three representative sections from three mice for each genotype.

### Establishment of primary cells from transgenic mice and growth as xenografts

Oligodendrocytes were purified from postnatal mice as described [55]. Lines were maintained in DMEM supplemented with 10% FCS (Omega Scientific, Tarzana, CA). 1×10^6^ cells were injected in the flanks of SCID (JAX Laboratories, Bar Harbor, ME) mice and growth assessed by direct caliper measurements.

### Protein analysis

Total protein from whole brain homogenates or cultured lines were extracted and subjected to SDS-PAGE, followed by transfer to PVDF membranes, blocked with 2.5% nonfat milk and incubated with primary antibody overnight at 4°C. The following primary antibodies were used in addition to those described above: Myc tag clone 9E10 (Millipore), Rictor, mTOR (Bethyl Laboratories, Montgomery, TX), phospho-S^32/36^-IkBα, IkBα, paxillin, phospho-Y^1068^-EGFR, EGFR (Cell Signaling), EGFR/EGFRvIII cocktail antibody (Upstate), anti-Myo1C (kind gift from Dr. Peter Gillespie, Vollum Institute, Oregon Health & Science University). Detection was performed using Amersham ECL (GE Healthcare, Piscataway, NJ). Immunoprecipitations were performed as previously described [19], utilizing normal mouse IgG (Millipore) as a non-specific control as indicated.

### Cell proliferation, clonogenic, cell migration and colony-forming assays

XTT assays (Roche, Indianapolis, IN) were used to assess cell proliferation in vitro. Cells were plated into 96-well plates at 1,000 per well and incubated for various time points. Cell numbers were subsequently determined by the addition of 2,3-bis[2-methoxy-4-nitro-5-sulfophenyl]-2H-tetrazolium-5-carboxanilide inner salt and assaying relative absorbance at 490 nm as described by the manufacturer. NIH3T3 and *Ink4a/Arf*-null MEF focus-forming assays were performed by plating 1×10^3^ cells per well in 24-well plates in a volume of 400 µL using a two-layered soft agar system as previously described [24]. The H-rasV^12^, Rictor and Myc expression vectors utilized in these assays have been previously described [24], [56], [57]. Cell migration assays were conducted using precoated modified Boyden chambers from Chemicon as recommended by the manufacturer and as previously described [24]. Invasion assays through Matrigel were performed by loading 2×10^5^ cells in the top well of Boyden chambers that contained growth factor-reduced Matrigel over a polyethylene terephthalate membrane with 8-mm pores (BD Biosciences, Franklin Lakes, NJ). Cells were allowed to invade and Matrigel removed prior to fixation and staining. Cells adhering to the bottom surface of the membrane were counted.

### SVZ measurements

Parasagittal sections from *GFAP-Cre/Rictor^loxP/loxP^ and GFAP-EGFRvIII; GFAP-Cre/Rictor^loxP/loxP^* mice, as well as, age matched littermate controls were aligned according to anatomical position based on hippocampal configuration and size. In this orientation a horizontal segment of the SVZ is displayed subjacent to the corpus callosum and an oblique segment connecting to a vertical section. The width of the SVZ at its widest point in the vertical section was measured perpendicular to the ependymal layer at x400 using a Nikon Optiphot-2® microscope equipped with a Optronics Microfire® digital camera. Optronics PictureFrame® imaging processing suite was used containing a calibrated scale bar for measurements. Mean + S.D. are shown and relative significant differences were determined using Student's *t* test.

## Supporting Information

Figure S1 **Rictor overexpression results in increased carcinogenesis.** (A) NIH3T3 focus forming assay in which cells were transfected with empty expression vector (vec), Rictor or c-myc containing expression vector DNA. (B) Cooperative increase in foci formation between Rictor and H-rasV^12^ in *Ink4a/Arf*-deficient MEFs. Data shown are mean + S.D. of three independent experiments.
(TIFF)

Figure S2 (**A**) **PCR amplification from tail clipped DNA showing the appropriate size bands for both transgenes of interest** (***GFAP-EGFRvIII*** **and** ***GFAP-Cre/Rictor^loxP/loxP^***) **in** ***GFAP-EGFRvIII; GFAP-Cre/Rictor^loxP/loxP^*** **transgenic mice.** (B) Immunoblot analysis comparing EGFRvIII-Y^1068^-phosphorylation, Rictor and Myo1C expression in NMO, Ric0 and R0E3 cells.
(TIFF)

Figure S3 (**A**) **H & E stained sections of xenografted Ric0 and R0E3 tumors from SCID mice.** 1×10^6^ cells were injected into the flanks of recipient mice and tumors harvested and sectioned for histological analysis. Arrows show oligodendroglial morphology of transplanted glioma cells derived from *GFAP-Cre/Rictor^loxP/loxP^* and *GFAP-EGFRvIII; GFAP-Cre/Rictor^loxP/loxP^* transgenic mice. Scale bar, 20 µm. (B) Cell extracts from NMO or Ric0 cells were immunoprecipitated with anti-mTOR antibodies and precipitates were immunobloted for the indicated proteins. Rictor demonstrates increased association with mTOR in Ric0 cells as compared to NMOs.
(TIFF)
