# Supplementary figures and images for: Conditional Astroglial Rictor Overexpression Induces Malignant Glioma in Mice
Source: PLoS One. 2012 Oct 15;7(10):e47741. doi: 10.1371/journal.pone.0047741 (PMC3471885; doi:10.1371/journal.pone.0047741)

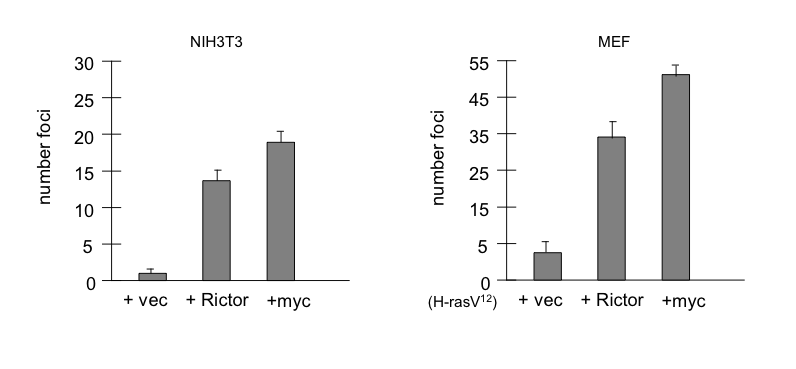

Supplement: Figure S1 — Rictor overexpression results in increased carcinogenesis. (A) NIH3T3 focus forming assay in which cells were transfected with empty expression vector (vec), Rictor or c-myc containing expression vector DNA. (B) Cooperative increase in foci formation between Rictor and H-rasV12 in Ink4a/Arf-deficient MEFs. Data shown are mean + S.D. of three independent experiments. (TIFF) [file pone.0047741.s001.tiff]

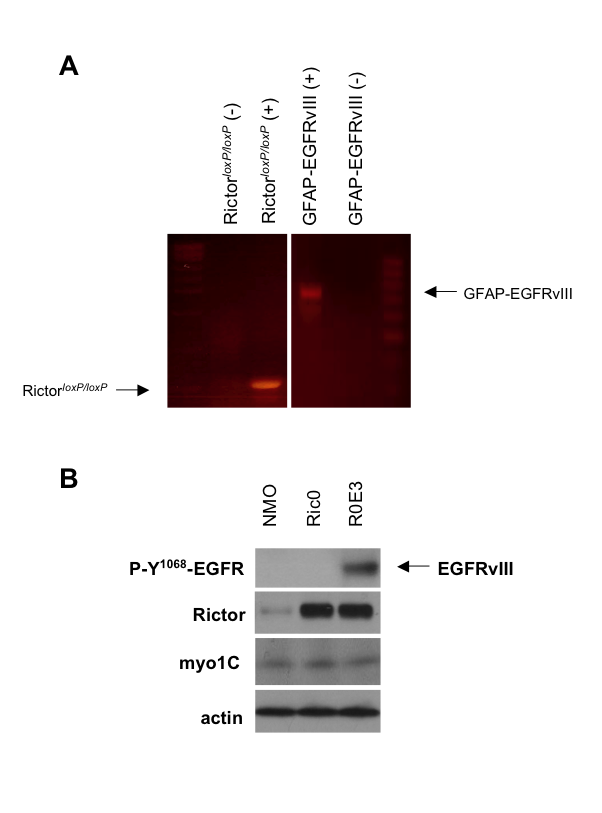

Supplement: Figure S2 — (A) PCR amplification from tail clipped DNA showing the appropriate size bands for both transgenes of interest (GFAP-EGFRvIII and GFAP-Cre/RictorloxP/loxP) in GFAP-EGFRvIII; GFAP-Cre/RictorloxP/loxP transgenic mice. (B) Immunoblot analysis comparing EGFRvIII-Y1068-phosphorylation, Rictor and Myo1C expression in NMO, Ric0 and R0E3 cells. (TIFF) [file pone.0047741.s002.tiff]

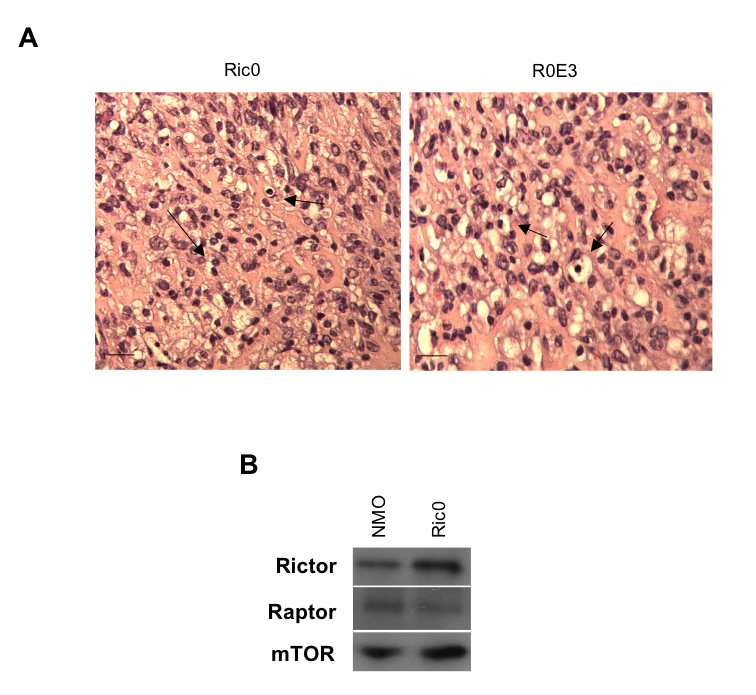

Supplement: Figure S3 — (A) H & E stained sections of xenografted Ric0 and R0E3 tumors from SCID mice. 1×10^6^ cells were injected into the flanks of recipient mice and tumors harvested and sectioned for histological analysis. Arrows show oligodendroglial morphology of transplanted glioma cells derived from GFAP-Cre/RictorloxP/loxP and GFAP-EGFRvIII; GFAP-Cre/RictorloxP/loxP transgenic mice. Scale bar, 20 µm. (B) Cell extracts from NMO or Ric0 cells were immunoprecipitated with anti-mTOR antibodies and precipitates were immunobloted for the indicated proteins. Rictor demonstrates increased association with mTOR in Ric0 cells as compared to NMOs. (TIFF) [file pone.0047741.s003.tiff]
